# Toxicity of Gold Nanoparticles in Mice due to Nanoparticle/Drug Interaction Induces Acute Kidney Damage

**DOI:** 10.1186/s11671-020-03371-4

**Published:** 2020-07-02

**Authors:** Katsuhiro Isoda, Anju Tanaka, Chisaki Fuzimori, Miyuki Echigoya, Yuichiro Taira, Ikuko Taira, Yoshimi Shimizu, Yoshihiro Akimoto, Hayato Kawakami, Isao Ishida

**Affiliations:** 1 Faculty of Pharmaceutical Sciences, Teikyo Heisei University, 4-21-2 Nakano-ku, Tokyo, 164-8530 Japan; 2 Department of Anatomy, Kyorin University School of Medicine, Mitaka, Tokyo 181-8611 Japan

**Keywords:** Gold nanoparticles, Kidney injury, Paraquat, Cisplatin, 5-Aminosalicylic acid

## Abstract

Nanomaterials are innovative materials with many useful properties, but there is concern regarding their many unknown effects on living organisms. Gold nanoparticles are widely used as industrial materials because of their excellent properties. The potential biological hazards of gold nanoparticles are unknown, and thus, here we examined the in vivo effects of gold nanoparticles 10, 50, and 100 nm in diameter (GnP10, GnP50, and GnP100, respectively) and their interactions with drugs in mice to clarify their safety in mammals. Cisplatin, paraquat, and 5-aminosalicylic acid cause side-effect damage to the liver and kidney in mice. No hepatotoxicity or nephrotoxicity was observed when any of the gold nanoparticles alone were administered via the tail vein. In contrast, co-administration of GnP-10 with cisplatin, paraquat, or 5-aminosalicylic acid caused side-effect damage to the kidney. This suggests that gold nanoparticles with a particle size of 10 nm are potentially nephrotoxic due to their interaction with drugs.

## Introduction

Nanotechnology is playing an increasingly important role in the twenty-first century, with nanomaterials underlying progress in nanotechnology. Recent developments in the manufacture of nanoparticles have aided the use of innovative nanomaterials worldwide [1, 2]. Nanomaterials have a diameter of 100 nm or less, and examples include gold, silver, silica, platinum, and titanium dioxide nanoparticles, as well as fullerenes and carbon nanotubes [3, 4]. These materials may find application in electronic storage technology, gene/regenerative medicine, and electronic devices, and are the foundations of new industries in the twenty-first century [5]. However, nanoparticles such as PM2.5 cause serious environmental pollution, respiratory diseases such as asthma, and ischemic heart disease [6]. Furthermore, diesel particles emitted by automobiles can have biological effects by entering the brain and reproductive organs [7], fibrous fine particles such as asbestos induce mesothelioma, and industrial fibrous nanomaterials such as carbon nanotubes may negatively impact human health [8, 9]. Many unknowns therefore remain regarding the biological effects of nanoparticles.

Gold (Au) has a low ionization tendency and high stability and has been used as a precious metal for decorative purposes since antiquity. Recently developed gold nanoparticles are widely used in medical and engineering applications due to their characteristic optical properties [10, 11], and their excellent optoelectronic properties have resulted in their use in organic solar cells, sensor probes, and conductive materials [12, 13]. Gold nanoparticles are used in the chemical industry as catalysts for acrylic resin synthesis. They also exhibit superior low-temperature catalytic activity for oxidizing CO compared to platinum nanoparticles, resulting in their use as an exhaust gas purification catalyst. Further applications of gold nanoparticles are expected in the future, but there has been little research on the toxicity of gold nanoparticles and their potential interaction with drugs.

The field of nanotechnology is expanding as researchers explore the safety, pharmacology, and pharmacokinetics of nanoparticles. Silica nanoparticles have been shown to cause cytotoxicity, hepatotoxicity, and placental damage [14, 15], and carbon nanotubes can induce pulmonary mesothelioma [16]. However, little is known of the pharmacological effects resulting from interactions between nanoparticles and drugs. In this study, we investigated the toxicity of gold particles 10, 50, and 100 nm in diameter (GnP10, GnP50, and GnP100, respectively) in mice to clarify their safety in mammals. In addition, we examined the effects of these nanoparticles on the toxicity of paraquat (PQ, a well-known hepatotoxin and nephrotoxin) [17], cisplatin (CDDP, a widely used antitumor agent) [18, 19], and 5-aminosalicylic acid (5-ASA, a common anti-inflammatory) [20].

## Results and Discussion

**Fig. 1:**
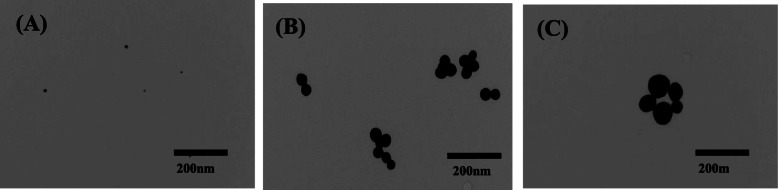
Ultrastructures of gold nanoparticles. Electron micrographs of GnP10 (**a**), GnP50 (**b**), and GnP100 (**c**) nanoparticles

We first measured the particle sizes of the gold nanoparticles using a Zetasizer, then observed the particles using transmission electron microscopy (Fig. 1a, b, c). The mean diameters of the GnP10, GnP50, and GnP100 nanoparticles were 15.7 ± 7.0, 53.3 ± 14.2, and 97.0 ± 27.1 nm, respectively (Supplemental Fig. 1). Furthermore, gold nanoparticles aggregate when measured by electron microscopy but do not aggregate when administered to mice. In addition, we measured the gold ion concentration by ICP-MS, but no ions were detected (data not shown). The surfaces of the gold nanoparticles were modified with citric acid to increase the affinity of the nanoparticles for water, but this modification exhibited no other functionality.

**Fig. 2:**
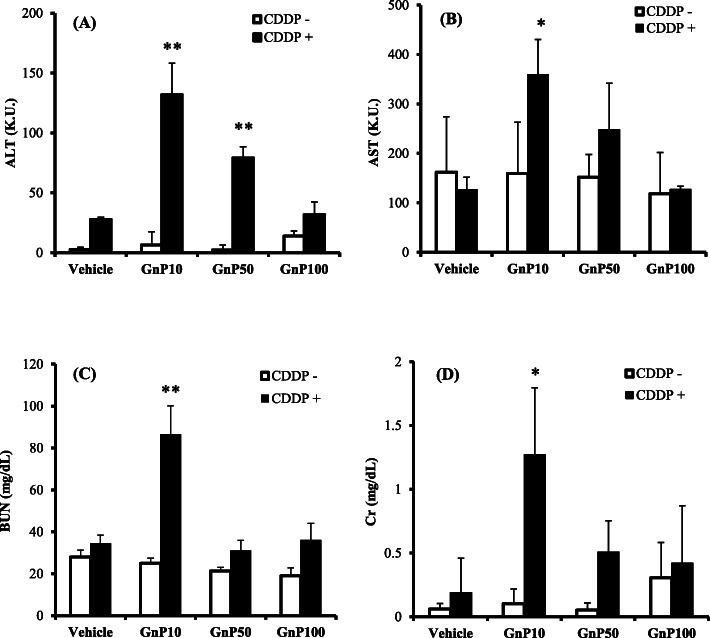
Effect of gold nanoparticles on cisplatin-induced toxicity. Mice were injected intraperitoneally with cisplatin (CDDP) at 0 (open bars) or 100 μmol/kg (solid bars), together with an IV injection of vehicle or gold nanoparticles (4 mg/kg). At 24-h post-injection, serum levels of the liver enzymes alanine aminotransferase (ALT; panel **a**) and aspartate aminotransferase (AST; panel **b**), and plasma levels of blood urea nitrogen (BUN; panel **c**), and creatinine (Cr; panel **d**) were determined using commercially available kits (see “Biochemical analyses” section). Data are presented as mean ± standard error of the mean (SEM; *n* = 4). Significant difference (*P < 0.05, **P < 0.01) between vehicle- and CDDP-treated groups

We examined whether GnP exhibits hepatotoxicity and nephrotoxicity by administering a maximum dose of 4 mg/kg to mice through the tail vein. No hepatotoxicity or nephrotoxicity was observed when gold nanoparticles alone were administered (Fig. 2). The ALT and AST values of mice administered GnP10, 50, and 100 alone (Fig. 2a, b) were similar to the control values, as were the BUN and Cr values. A single administration of GnP to mice did not induce liver or kidney damage, nor heart, lung, or spleen damage (Supplemental Fig. 2), indicating that gold nanoparticles are non-toxic when administered alone to mice.

**Fig. 3:**
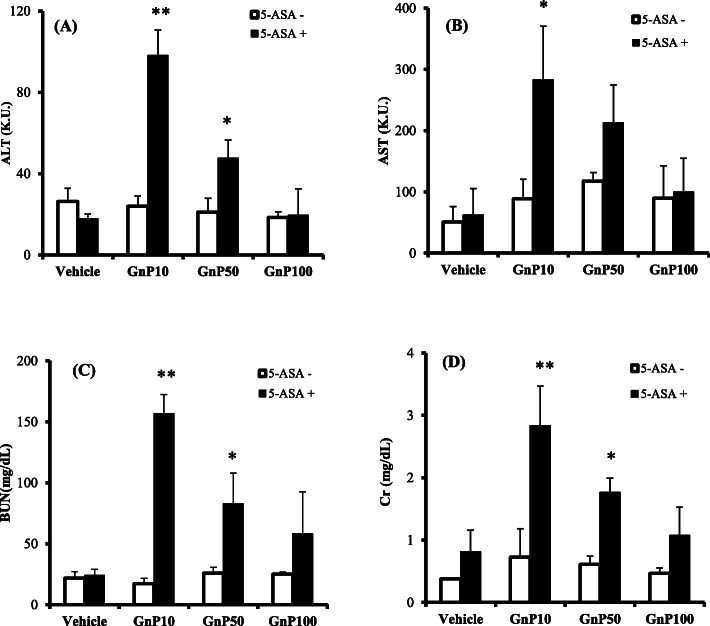
Effect of gold nanoparticles on 5-aminosalicylic acid-induced toxicity. Mice were injected intraperitoneally with 5-aminosalicylic acid (5-ASA) at 0 (open bars) or 500 mg/kg (solid bars), together with an IV injection of vehicle or gold nanoparticles (4 mg/kg). At 24-h post-injection, serum levels of the liver enzymes alanine aminotransferase (ALT; **a**) and aspartate aminotransferase (AST; B), and plasma levels of blood urea nitrogen (BUN; **c**) and creatinine (Cr; **d**) were determined using commercially available kits (see “Biochemical analyses” section). Data are presented as mean ± standard error of the mean (SEM; *n* = 4). Significant difference (*P < 0.05, **P < 0.01) between vehicle- and 5-ASA-treated groups

**Fig. 4:**
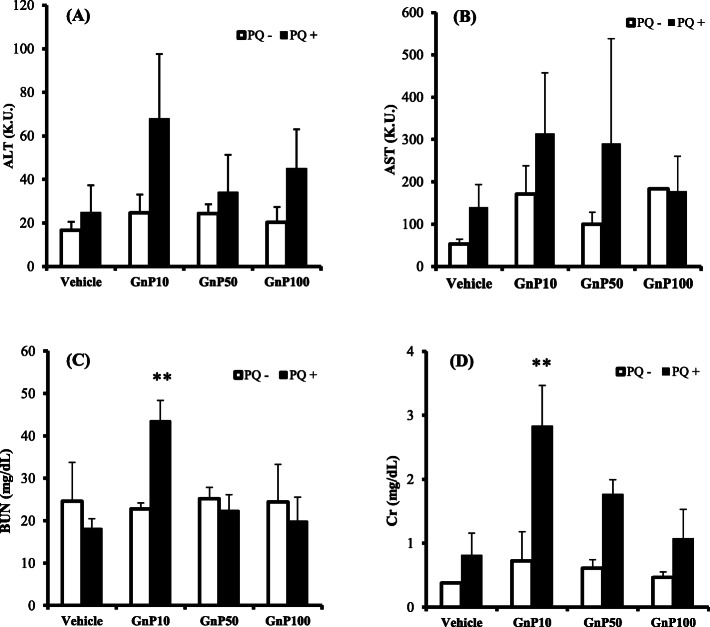
Effect of gold nanoparticles on paraquat-induced toxicity. Mice were injected intraperitoneally with paraquat (PQ) at 0 (open bars) or 50 mg/kg (solid bars), together with an IV injection of vehicle or gold nanoparticles (4 mg/kg). At 24-h post-injection, serum levels of the liver enzymes alanine aminotransferase (ALT; **a**) and aspartate aminotransferase (AST; **b**), and plasma levels of blood urea nitrogen (BUN; **c**) and creatinine (Cr; **d**) were determined using commercially available kits (see “Biochemical analyses” section). Data are presented as mean ± standard error of the mean (SEM; *n* = 4). Significant difference (*P < 0.05, **P < 0.01) between vehicle- and PQ-treated groups

Liver and kidney damage has been reported to be induced by the co-administration of silica nanoparticles, nanoclays, or polystyrene nanoparticles with drugs or chemicals [14, 21, 22]. We therefore co-administered gold nanoparticles with PQ (a liver-kidney toxin) or the drugs CDDP or 5-ASA (which cause adverse liver-nephrotoxic effects). Figure 2 shows the results of the interaction between gold nanoparticles and CDDP. The co-administration of GnP10 or GnP50 and CDDP increased ALT and induced liver damage (Fig. 2a), and the co-administration of GnP10 and CDDP increased BUN and Cr, inducing renal damage (Fig. 2c, d). We then investigated the interaction between GnP and 5-ASA, a widely used anti-inflammatory drug that causes liver and kidney damage. The co-administration of GnP10 or GnP50 with CDDP increased ALT and induced liver damage (Fig. 3a), whereas co-administration with 5-ASA increased BUN and Cr and induced renal damage (Fig. 3c, d). Next, we investigated the interaction between GnP and PQ, a widely used agrochemical that causes liver and kidney damage. The co-administration of GnP10 and PQ increased BUN and Cr levels and induced renal damage (Fig. 4c, d) but not liver damage (Fig. 4a, b). The co-administration of the smallest gold particles tested, GnP10, with CDDP, 5-ASA, or PQ resulted in the highest ALT, BUN, and Cr values observed in this study. The 10× larger particles of GnP100 did not cause liver or kidney damage when co-administered with CDDP, 5-ASA, or PQ. These results show that GnP is toxic when particles less than 100 nm in diameter are co-administered with CDDP, 5-ASA, or PQ.

**Fig. 5:**
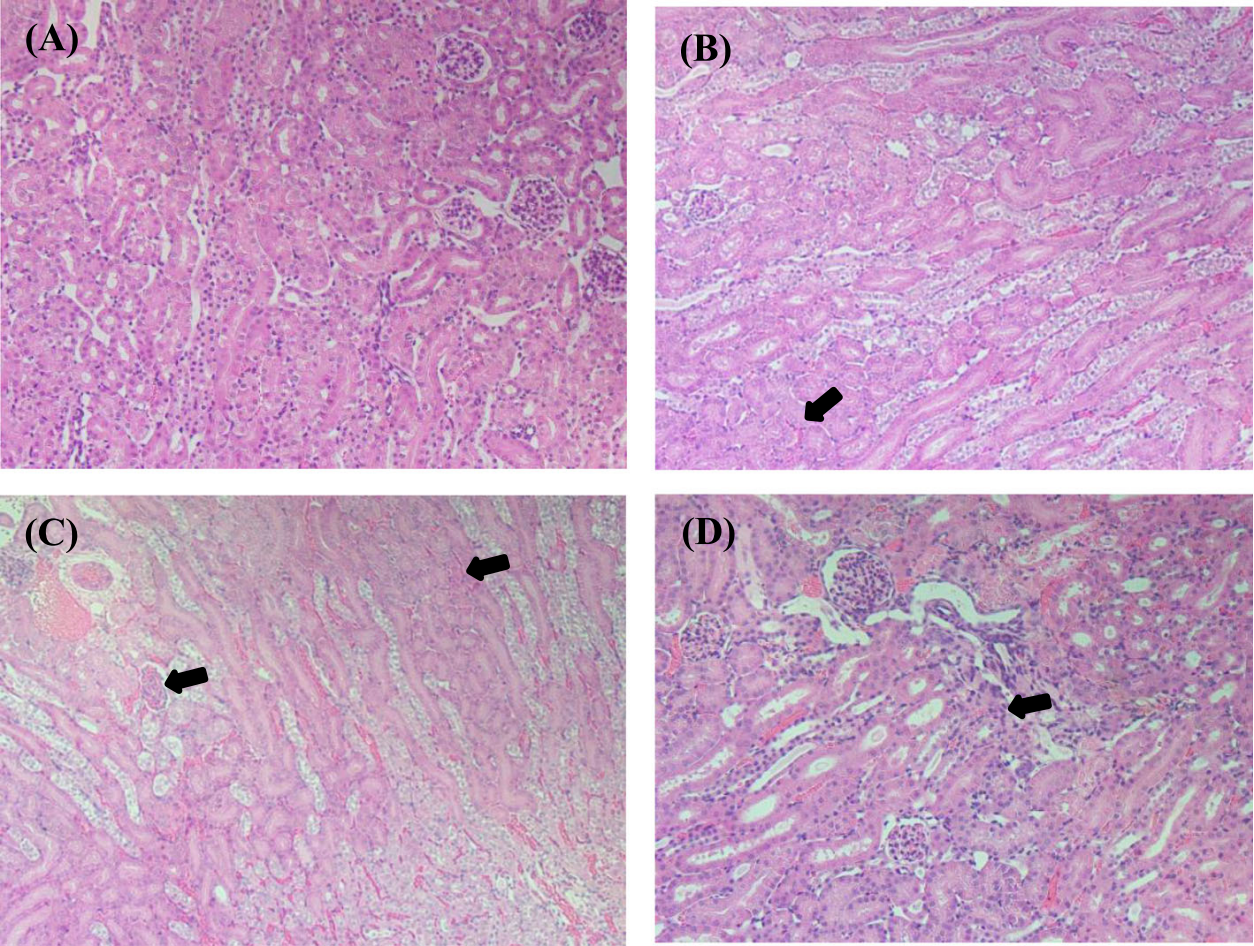
Histologic analysis of kidney tissues from gold nanoparticle–treated mice. At 24-h post-IV administration of only GnP10 (**a**), GnP10 with CDDP (**b**), GnP10 with 5-ASA (**c**), and GnP10 with PQ (**d**), tissues were collected, fixed with 4% paraformaldehyde, sectioned, and stained with hematoxylin and eosin. Arrows designate sites of kidney damage

**Fig. 6:**
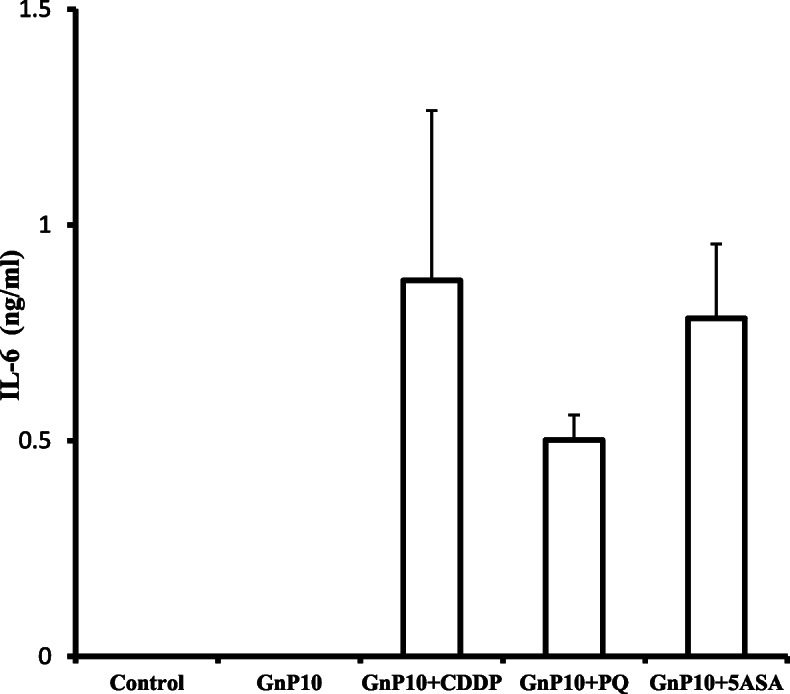
IL-6 levels in serum, as measured by ELISA. Mice received an IV injection of GnP10 with CDDP, 5-ASA, or PQ. Cytokine levels were measured 3 h after administration. Values are the mean ± standard error (SE; *n* = 4)

Renal hematoxylin and eosin observation following co-administration of GnP10 with CDDP, 5-ASA, or PQ (Fig. 5) showed tubule damage, suggesting the induction of acute kidney injury. Next, we measured IL-6 and TNF-α in serum to investigate the underlying cause of GnP10-induced acute kidney damage. Figure 6 shows serum IL-6 levels 3 h after the co-administration of GnP10 with CDDP, 5-ASA, or PQ. IL-6 was not detected in the GnP10-alone group, but an increase in IL-6 was observed when GnP10 was co-administered with CDDP, 5-ASA, or PQ. TNF-α was not detected in any group (data not shown). These results suggest that IL-6 is involved in acute kidney damage induced by GnP10 and CDDP, 5-ASA, or PQ.

We investigated the effect of co-administering gold nanoparticles and drugs on side effects (i.e., liver and kidney damage). The smallest particle size, GnP10, induced kidney and liver damage upon co-administration with CDDP, 5-ASA, or PQ. We also co-administered GnP10 with acetaminophen, streptomycin, or tetracycline to mice and observed no liver or kidney damage (data not shown). We previously reported that silica nanoparticles induce liver damage, depending on the particle size [23], and polystyrene nanoparticles may induce liver damage upon co-administration with a drug, depending on the particle size [24]. Xia et al. reported that smaller gold nanoparticles are more genotoxic in vitro [25]. Taken together, gold nanoparticles become highly toxic due to interactions with drugs as the particle size is reduced.

Co-administration of Gnp10 with cisplatin, 5-ASA, or PQ increased IL-6 levels (Fig. 6). IL-6 was not elevated by GnP10 administration alone (Fig. 6) or by the administration of CDDP, PQ, or 5-ASA alone (data not shown). IL-6 was previously reported to be involved in the induction of liver [26] and acute kidney [27, 28] damage. We believe that Gnp10 induces IL-6, which in turn induces liver and kidney damage, but the underlying mechanism remains unclear. Bauza et al. report that IL-6 induces liver damage by inducing transcription factors in hepatocytes [29]. Understanding the involvement of cell-specific transcription factors in IL-6-induced liver and kidney damage will require further experiments on the mechanism behind the cytotoxicity of gold nanoparticles.

Recently, gold nanoparticles have attracted attention as a functional biomaterial used in drug delivery systems [30], and research on cancer treatment using gold nanoparticles is being actively conducted. For example, Anselmo et al. reported that PEG-coated silica-gold nanoparticles increased the local temperature upon absorbing light and thermally dissolved solid tumors [31], showing that gold nanoparticles are promising materials for cancer treatment. However, we found that interaction between the anticancer drug cisplatin and gold nanoparticles induced renal damage (Fig. 2), suggesting that the use of gold nanoparticles in cancer treatment requires study of their safety when co-administered with the drug.

## Conclusions

In summary, Gnp10 caused kidney damage upon co-administration with CDDP, PQ, or 5-ASA. GnP50 caused kidney damage only when co-administered with 5-ASA whereas GnP100 did not. We demonstrated that gold nanoparticles can cause kidney damage and that this effect can be synergistically exacerbated as a result of interactions with chemicals or drugs. Further studies based on these data will be required to fully elucidate the toxicological profiles of nanoparticles proposed for diagnostic or therapeutic use.

## Materials and Methods

### Materials

Suspensions of citrate-ligand-capped gold particles 10, 50, and 100 nm in diameter were obtained from NANOCOMPOSIX, INC. (San Diego, CA, USA). The size distributions of the particles were analyzed using a Zetasizer (Sysmex Co., Kobe, Japan) and a TEM JEOL JEM-1011 transmission electron microscope. The mean diameters were 15.7 ± 7.0, 53.3 ± 14.2, and 87.0 ± 27.1 nm (Fig. 1, Supplemental Fig. 1). Aqueous suspensions (1 mg/mL) were thoroughly dispersed by sonication before use and diluted with water. The presence of ionized gold in the gold nanoparticle suspensions was examined by ICP-MS, and no ionized gold was detected. Identical volumes of each suspension were injected into mice for each experiment. The geometric sizes of the particles were characterized by TEM. Paraquat (Sigma-Aldrich, St. Louis, MO, USA), cisplatin, and 5-aminosalicylic acid (Wako Pure Chemical Industries, Osaka, Japan) were dissolved in saline and stored at − 20 °C until use. All reagents were research grade.

### Animals

Eight-week-old BALB/c male mice were purchased from Funabashi Farm Co., Ltd. (Chiba, Japan). The animals were maintained in a controlled environment (temperature 23 ± 1.5 °C; light 12-h light/dark cycle) with free access to standard rodent chow and water. The mice were given 1 week to acclimate before initiating the experiments. The experimental protocols conformed to the ethical guidelines of the Teikyo Heisei University Graduate School of Pharmaceutical Sciences, compiled from the Guidelines for Animal Experimentation of the Japanese Association for Laboratory Animal Sciences.

### Biochemical Analyses

Serum alanine aminotransferase (ALT), serum aspartate aminotransferase (AST), blood urea nitrogen (BUN), and creatinine (Cr) were measured using commercially available kits (Wako Pure Chemical Industries) according to the manufacturer’s protocols. Briefly, collected serum (10 mL) was combined with 1 mL of color A reagent (containing urease) and incubated at 37 °C for 15 min. Following the addition of 1 mL of color B reagent, the sample was incubated at 37 °C for 10 min. Absorbance was measured at a wavelength of 570 nm. Interleukin (IL)-6 and TNF-α were analyzed using an enzyme-linked immunosorbent assay (ELISA) kit (BioSource International, CA, USA). All analyses were performed in strict accordance with the manufacturer’s instructions.

### Histological Analysis

At 24 h after dose administration, the animals were sacrificed, and the livers were removed and fixed with 4% paraformaldehyde. Following processing and sectioning, thin tissue sections were stained with hematoxylin and eosin for histological observation.

### Statistical Analysis

Statistical analyses were performed with the Statcel add-in forms, 3rd Excel Software (EMS publication Co., Ltd., Saitama, Japan). All data are presented as means ± standard error of the mean (SEM). Significant differences between the control and experimental groups were determined using the Dunnett test; a *P* value less than 0.05 was considered significant.

## Additional Files

**Additional file 1: Figure S1.** Results of gold nanoparticle diameter measurement. A) GnP10. The average GnP10 diameter indicated by the peak was 15.7 ± 7.0 nm. B) GnP50. The average GnP50 diameter indicated by the peak was 53.3 ± 14.2 nm. C) GnP100. The average GnP100 diameter indicated by the peak was 87.0 ± 27.1 nm. **Figure S2.** Histologic analysis following a single administration of GnP to mice. At 24-h post-IV administration of only GnP10, Gnp50 or GnP100, the tissues were collected, fixed with 4% paraformaldehyde, sectioned, and stained with hematoxylin and eosin. The liver, heart, kidneys, lungs and spleen were observed.

## Data Availability

Not applicable.

## Abbreviations

ALT: Alanine aminotransferase
AST: Aspartate aminotransferase
BUN: Blood urea nitrogen
PQ: Paraquat
Cr: Creatinine
