# Supplementary figures and images for: Toxicity of Gold Nanoparticles in Mice due to Nanoparticle/Drug Interaction Induces Acute Kidney Damage
Source: Nanoscale Res Lett. 2020 Jul 2;15:141. doi: 10.1186/s11671-020-03371-4 (PMC7332653; doi:10.1186/s11671-020-03371-4)

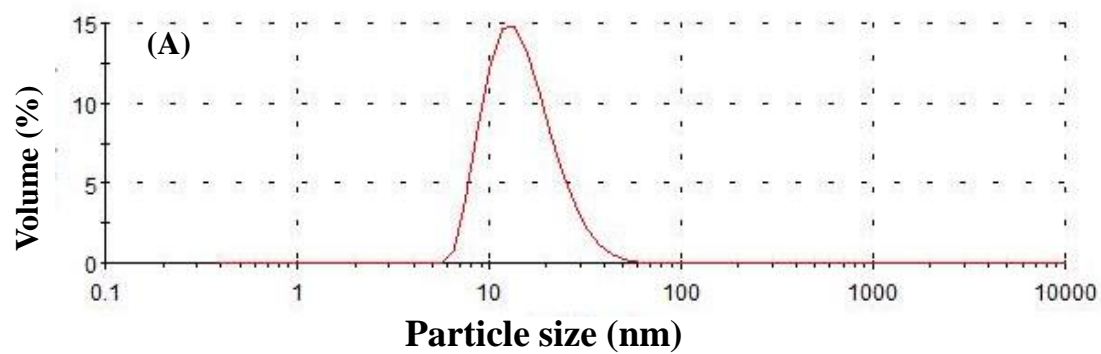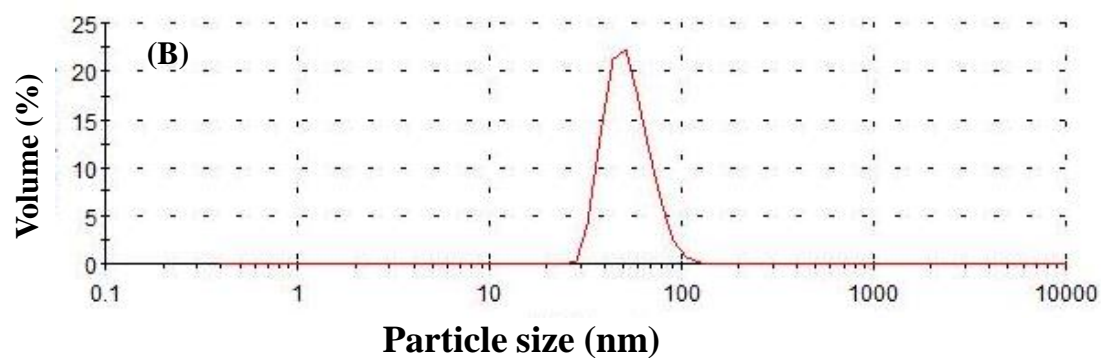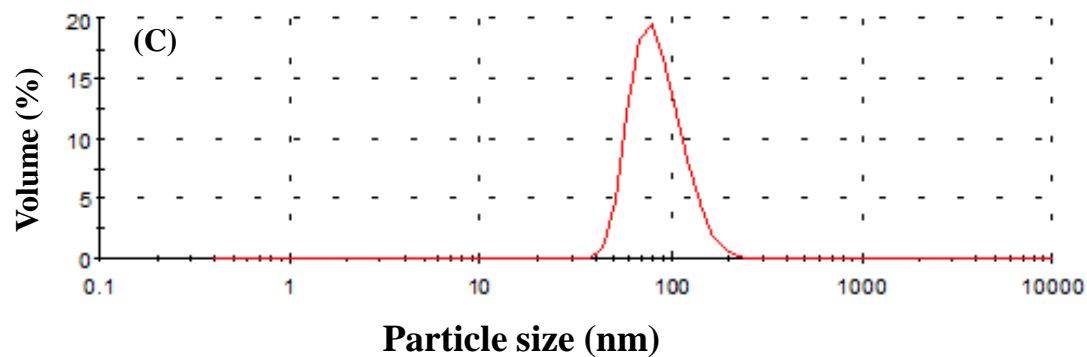

Supplementary Fig. 1

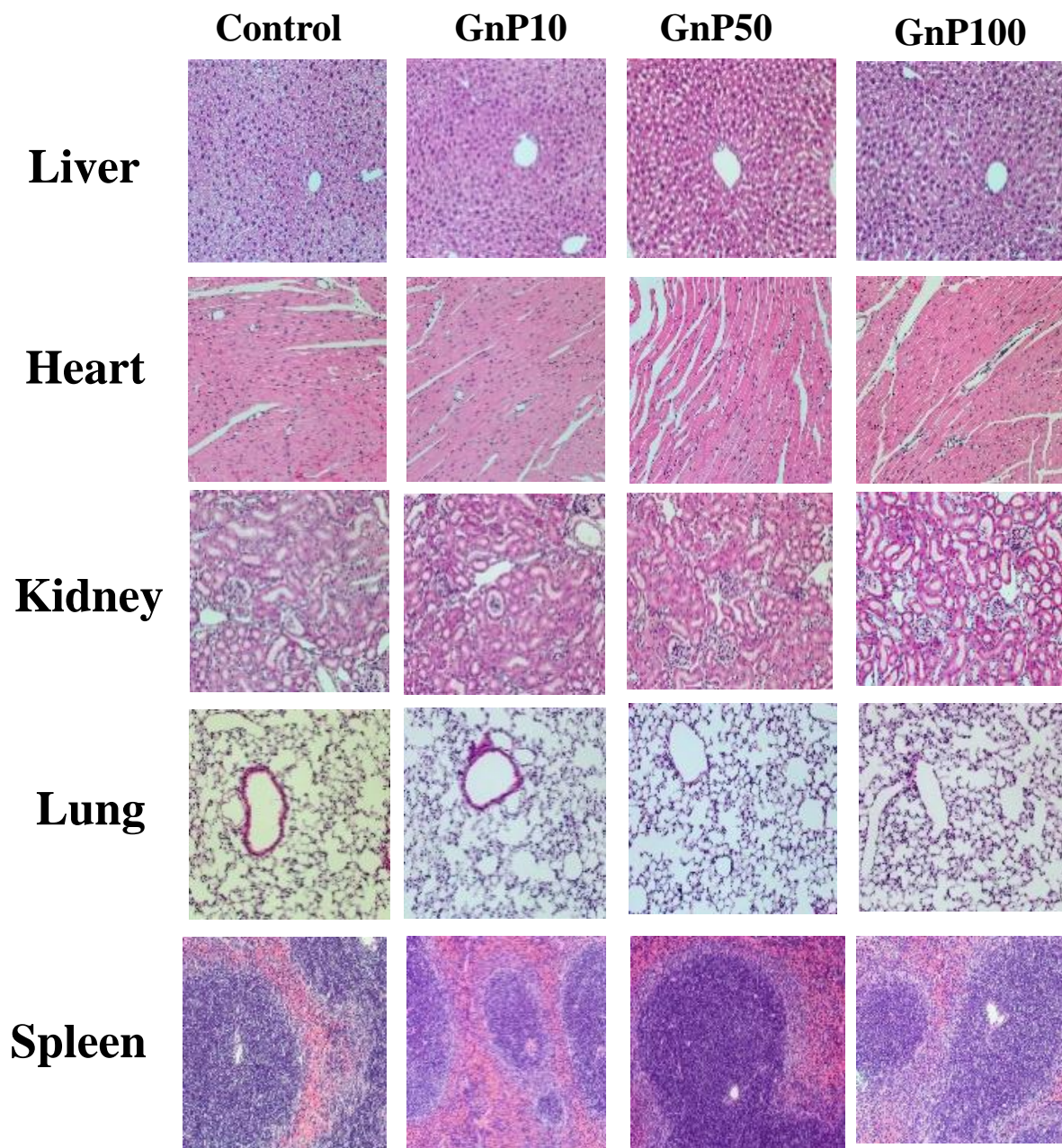

Supplementary Fig. 2

Supplement: Supplementary file 1 — Additional file 1: Figure S1. Results of gold nanoparticle diameter measurement. A) GnP10. The average GnP10 diameter indicated by the peak was 15.7 ± 7.0 nm. B) GnP50. The average GnP50 diameter indicated by the peak was 53.3 ± 14.2 nm. C) GnP100. The average GnP100 diameter indicated by the peak was 87.0 ± 27.1 nm. Figure S2. Histologic analysis following a single administration of GnP to mice. At 24-h post-IV administration of only GnP10, Gnp50 or GnP100, the tissues were collected, fixed with 4% paraformaldehyde, sectioned, and stained with hematoxylin and eosin. The liver, heart, kidneys, lungs and spleen were observed. [file 11671_2020_3371_MOESM1_ESM.pdf]
